# Utility of ^99m^ Technetium Pertechnetate Thyroid Scan and Uptake in Thyrotoxic Patients: Jordanian Experience

**DOI:** 10.1055/s-0042-1751053

**Published:** 2022-09-02

**Authors:** Kusai M. Al-Muqbel

**Affiliations:** 1 Faculty of Medicine, Department of Radiology and Nuclear Medicine, Jordan University of Science and Technology, Irbid, Jordan

**Keywords:** thyroid uptake, technetium-99m, thyrotoxicosis, radioiodine, Graves' disease, toxic nodular goiter

## Abstract

**Objective** The objective of this study was to assess our local experience with ^99m^ Technitium thyroid uptake (TcTU) in thyrotoxicosis by examining mean and range of TcTU in both euthyroid patients and thyrotoxic patients. We also wanted to see how well TcTU performed as a substitute for radioiodine thyroid uptake in thyrotoxicosis.

**Methods** The medical records of thyrotoxic patients were reviewed retrospectively. Inclusion criteria were: (1) thyrotoxicosis was proven biochemically, (2) the patient underwent TcTU at the time of thyrotoxicosis diagnosis, (3) at least 6 months of follow-up, and (4) the final diagnosis was documented. All TcTU of euthyroid patients were also reviewed to determine local normal TcTU mean and range. Patients were divided into three groups: Graves' disease, toxic nodular goiter, and subacute thyroiditis. Each patient group's TcTU mean and range were assessed separately.

**Results** There were 209 patients in total (54 euthyroid, 112 Graves' disease, 26 toxic nodules, and 17 patients with subacute thyroiditis). TcTU mean±standard deviation and range for euthyroid patients were 1.5±1.1% and 0.17 to 4.8%, 10.6±10% and 0.43 to 40% for Graves' disease, 4.5±4% and 0.6 to 15% for toxic nodules, and 0.5±0.4% and 0.18 to 1% for subacute thyroiditis. Although one-third of thyrotoxic patients' TcTU values overlapped with the normal TcTU range, the diagnosis was made using qualitative image analysis. Subacute thyroiditis was characterized by poor thyroid visualization, whereas Graves'/toxic nodular goiter was well visualized.

**Conclusion** The mean and range of our local normal TcTU were similar to those previously published. TcTU was a useful alternative to radioiodine in the evaluation of thyrotoxicosis. About two-thirds of the patients had accurate test results. The diagnosis was reached in the remaining one-third of patients by combining quantitative and qualitative image features. This method allowed us to stop giving radioiodine to our patients, resulting in a significant reduction in patient radiation exposure.

## Introduction

Thyrotoxicosis is a common endocrine medical condition characterized by a variety of nonspecific symptoms (including but not limited to tremor, palpitation, excessive sweating, weight loss, diarrhea, nervousness). Thyroid-stimulating hormone (TSH) is suppressed in thyrotoxic patients, who have elevated thyroid hormones (T3 and T4). Subacute thyroiditis, Graves' disease, and toxic nodular goiter are all causes of thyrotoxicosis. The thyroid scan and uptake nuclear test can differentiate between thyrotoxicosis causes. 1 2 Because of its low cost, ready availability, rapid single-visit imaging, lower radiation burden on the patient, and good image quality, ^99m^ Technetium ( ^99m^ Tc) pertechnetate is used in thyroid uptake evaluation. 1 Radioiodine-123 ( ^123^ I) is expensive and requires a return visit by the patient the next day. 1 Radioiodine-131 ( ^131^ I) has a high radiation burden on the patients so it is abandoned in developed countries. 2 As a result, ^99m^ Tc pertechnetate is used in our laboratory for thyroid scans and uptake, while ^131^ I has been abandoned for several years.

^99m^ Tc thyroid uptake (TcTU), on the other hand, has a narrow normal uptake range of 0.5 to 4%, which overlaps with the ranges of thyrotoxic patients. 3 4 As a result, TcTU values are sometimes considered inaccurate in thyrotoxic patients.

The aim of this study was to assess our local experience with TcTU in thyrotoxicosis by examining our normal TcTU mean and range, as well as the mean and range of TcTU in each thyrotoxic patient group. We also wanted to see how well TcTU performed as a substitute for radioiodine thyroid uptake in the evaluation of thyrotoxicosis.

## Materials and Methods

From March 2016 to December 2020, the medical records of thyrotoxic patients were reviewed retrospectively. The hospital's institutional review board (IRB) has given its approval to the study (Reference # 5/118/2018 date August 26, 2018). The need for written informed consent was waived. Inclusion criteria were: (1) thyrotoxicosis proven biochemically by thyroid function test (TFT) including suppressed TSH with or without elevated T3 and T4, (2) the patient underwent TcTU and scan at the time of thyrotoxicosis diagnosis, (3) follow-up was continuous in our hospital for at least 6 months after obtaining TcTU and scan, and (4) the final diagnosis is established in the medical record after at least 6 months of follow-up. All tests of TcTU and scan of euthyroid patients referred for thyroid nodule evaluation were retrospectively reviewed from March 2016 to December 2020 to determine our local normal TcTU reference mean and range. If the test of TcTU and scan was performed within a week of obtaining a normal TFT, the euthyroid patient was included in the study. Patients were divided into three groups based on their final diagnosis in the medical records: Graves' disease, toxic nodular goiter (single or multiple), and subacute thyroiditis. TcTU mean and range were evaluated retrospectively for each group separately.

### Technique

#### ^99m^ Tc Thyroid Scintiscan

Twenty minutes after intravenous administration of 18.5-MBq (5 mCi) ^99m^ Tc pertechnetate, 5-minute static thyroid images were obtained in anterior, right anterior oblique, and left anterior oblique views while the patient is supine with an extended neck. Images were obtained by a large-field-of-view gamma camera (Mediso, AnyScan, Budapest, Hungary) fitted with low-energy all-purpose parallel-hole collimator. Image data were recorded on a 128×128 computer matrix.

#### ^99m^ Tc Thyroid Uptake Measurement

TcTU was obtained utilizing MEDISO thyroid uptake software. Data required by the software were: injected dose measured by MBq in the syringe and time of measurement, syringe residual dose measured by MBq and time of measurement, thyroid region of interest (ROI), and neck background ROI.

## Results

Our study included 219 patients, with 155 thyrotoxic patients and 54 euthyroid patients. Females made up 72% of the patients, while males made up 28%. Most of the patients were middle-aged, an average±standard deviation (SD) of 43±15 years and a range of 7 to 81 years ( Table 1 ). Table 2 shows the mean±SD TFT results in 155 thyrotoxic patients.

**Table 1:** Number, age, and gender of patients who underwent ^99m^ Tc thyroid scan and uptake, and classified as normal (euthyroid), hyperthyroid (Graves' disease or toxic nodules), or subacute thyroiditis

Normal (euthyroid)		Graves' disease		Toxic nodules		Subacute thyroiditis		Total	
No. (F:M)	Age (y) range (mean±SD)	No. (F:M)	Age (y) range (mean±SD)	No. (F:M)	Age (y) range (mean±SD)	No. (F:M)	Age (y) range (mean±SD)	
54 (38:16)	7–78 (44±16)	112 (79:33)	15–72 (40±13)	26 (20:6)	20–81 (46±15)	17 (14:3)	22–73 (39±15)	209

Abbreviations: F, female; M, male; SD, standard deviation; y, years.

**Table 2:** Mean±SD of thyroid function test results in patient groups, including local laboratory normal reference range

	Normal (euthyroid)	Graves' disease	Toxic nodules	Subacute thyroiditis	Reference range
TSH mIU/L	1.9±1.2	0.011±0.017	0.025±0.33	0.019±0.016	0.27–4.64
T4 pmol/L	16±6.5	49±37	27±12	33±22	12–22
T3 pmol/L	5±1.6	19±13	10±5	9±4	3.1–6.8

Abbreviations: SD, standard deviation; TSH, thyroid-stimulating hormone.

**Fig. 1:**
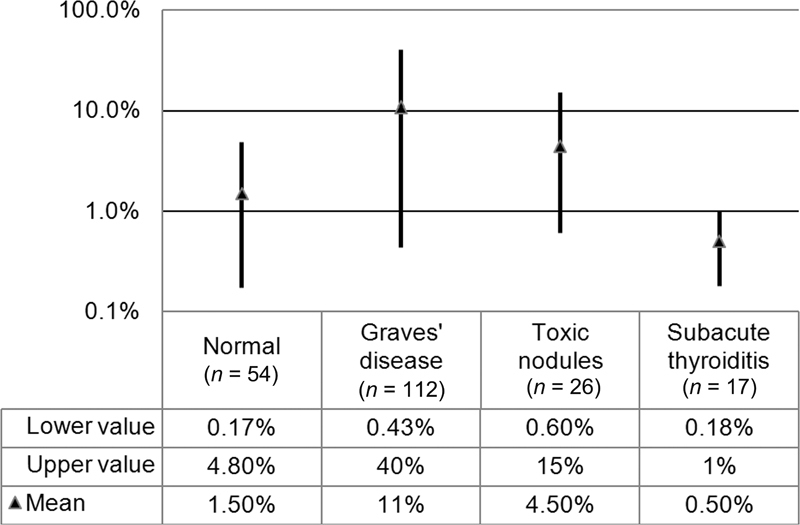
Mean and range of ^99m^ Tc thyroid uptake (TcTU) in euthyroid (normal) and thyrotoxic (Graves' disease, toxic nodular goiter, and subacute thyroiditis) groups. Overlap is demonstrated between normal TcTU range and TcTU ranges of thyrotoxic patient groups. The standard deviation values are 1.1%, 10, 4, and 0.4% in the normal, Graves' disease, toxic nodular goiter, and subacute thyroiditis groups, respectively.

**Fig. 2:**
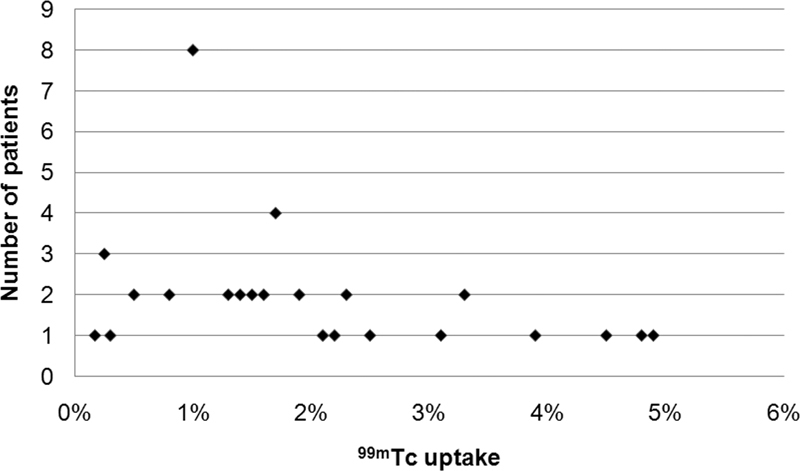
Scatter plot of ^99m^ Technetium ( ^99m^ Tc) thyroid uptake values in the normal (euthyroid) group (54 patients).

**Fig. 3:**
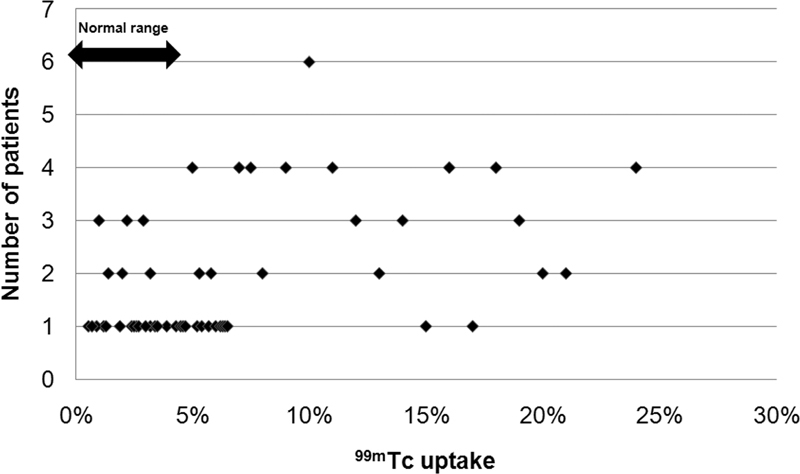
Scatter plot of ^99m^ Technetium ( ^99m^ Tc) thyroid uptake values in the Graves' disease group (54 patients), with some values falling within the normal range.

**Fig. 4:**
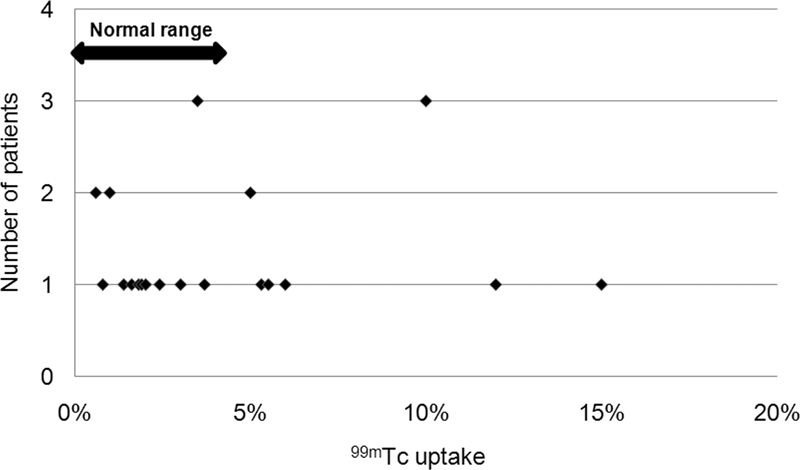
Scatter plot of ^99m^ Technetium ( ^99m^ Tc) thyroid uptake values in toxic nodules group (26 patients), with some values falling within the normal range.

**Fig. 5:**
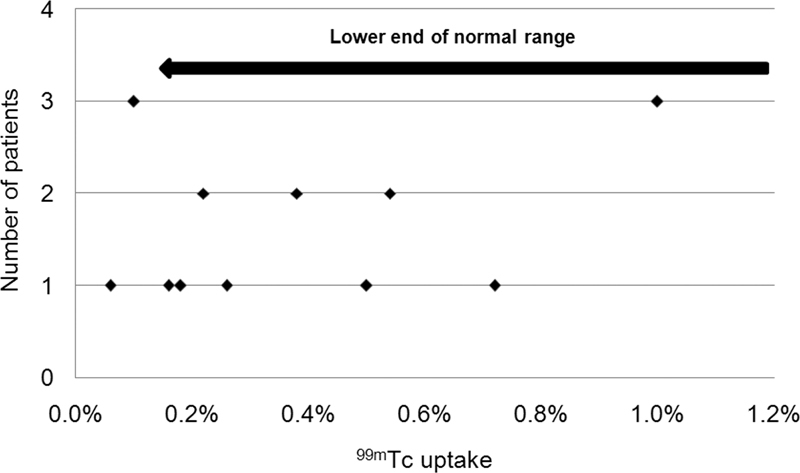
Scatter plot of ^99m^ Technetium ( ^99m^ Tc) thyroid uptake values in subacute thyroiditis group (17 patients), with some values falling within the normal range.

Fig. 1 summarizes TcTU mean±SD and range in each patient group. In the euthyroid group, the normal TcTU mean±SD and range were 1.5±1.1% and 0.17 to 4.8%, respectively ( Fig. 2 ). The TcTU mean was significantly higher in the Graves' disease and toxic nodules groups, while it was significantly lower in the subacute thyroiditis group ( Fig. 1 ). The mean±SD and range of TcTU in the Graves' disease group were 10.6±10% and 0.43 to 40%, respectively ( Fig. 3 ). TcTU mean±SD and range for the toxic nodules group were 4.5±4% and 0.6 to 15%, respectively ( Fig. 4 ). Subacute thyroiditis TcTU mean±SD and range were 0.5±0.4% and 0.18 to 1%, respectively ( Fig. 5 ).

**Fig. 6:**
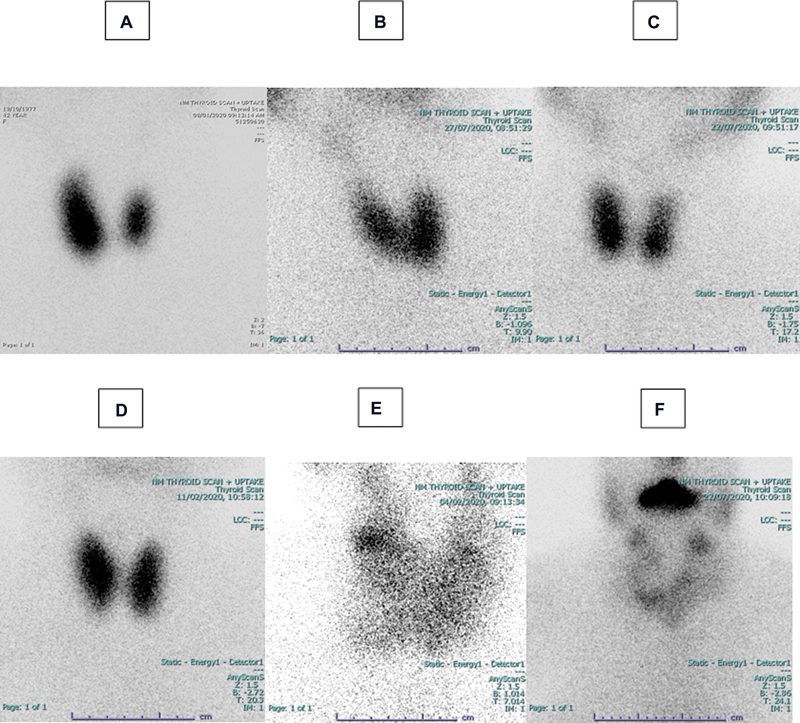
Thyroid scans with ^99m^ Technetium ( ^99m^ Tc) pertechnetate in four patients with Graves' disease ( **A, B, C,** and **D** ) and two patients with toxic nodular goiter ( **E** and **F** ). Patient A has a high ^99m^ Tc uptake value of 9%, whereas patients **B** , **C** , and **D** , respectively, have normal ^99m^ Tc uptake values of 2.5, 1.2, and 3%. The thyroid gland, on the other hand, is clearly visible in all of them. Despite having a toxic multinodular goiter with normal ^99m^ Tc uptake values of 2 and 1%, respectively, patients **E** and **F** have well-visualized hot nodules.

**Fig. 7:**
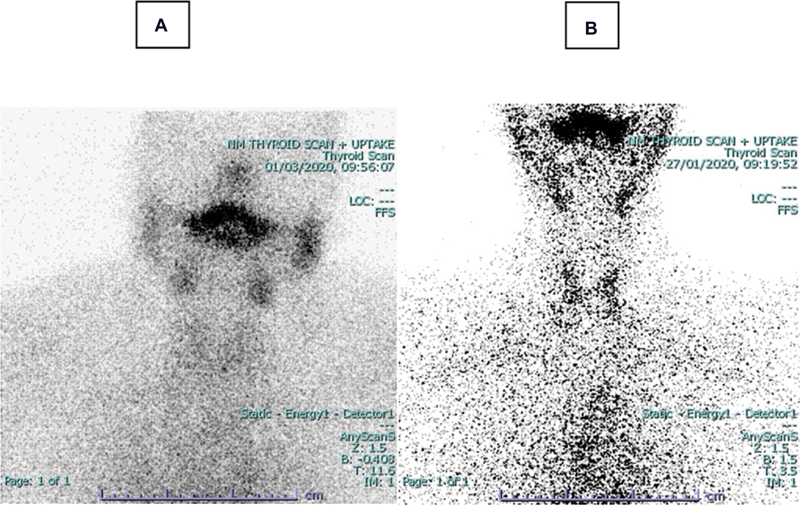
Thyroid scans with ^99m^ Technetium ( ^99m^ Tc) pertechnetate in two patients with subacute thyroiditis. Both patients have extremely low ^99m^ Tc uptake values, with 0.1% in patient **A** and 0.2% in patient **B** . At the same time, thyroid gland is poorly visualized in both patients.

TcTU values were normal in 31% (49/155) of thyrotoxic patients (overlapped with TcTU normal range). However, whether TcTU is normal or high, goiter in Graves' disease and hot nodules in toxic nodular goiter were well visualized ( Fig. 6 ). In subacute thyroiditis, whether TcTU values were low or low normal, poor visualization of the thyroid gland was observed ( Fig. 7 ).

## Discussion

Thyroid uptake value combined with thyroid scintigraphic features can help determine the cause of thyrotoxicosis. Subacute thyroiditis is frequently associated with reduced uptake value and poor thyroid gland visualization, whereas Graves' disease or a toxic nodular goiter is frequently associated with elevated uptake value and diffusely enlarged thyroid or focal nodules. 1 2

Because of its low cost, ready availability, rapid single-visit imaging, lower radiation burden on the patient, and good image quality, ^99m^ Tc pertechnetate is used in thyroid uptake evaluation. 1 Thyroid uptake of ^99m^ Tc pertechnetate is closely correlated with radioiodine uptake in thyrotoxic patients, despite the fact that ^99m^ Tc pertechnetate does not undergo organification by follicular cells like iodine. 5 ^123^ I produces high-quality images while exposing the patient to small amount of radiation. However, ^123^ I is expensive and not widely available, and the test requires the patient to return the next day. 1 Because of its high radiation burden on patients due to its long half-life and β-radiation, ^131^ I has been abandoned in Europe and North America. 2 As a result, we have been using ^99m^ Tc pertechnetate instead of ^131^ I for TcTU and scanning in our laboratory since 2015. The purpose of this retrospective study is to reflect our experience with the use of ^99m^ Tc pertechnetate in the evaluation of our thyrotoxic patients.

Our local normal TcTU ranged from 0.17 to 4.8% in the current study, with a mean±SD of 1.5±1.1%. Our findings are very similar to those previously published. Atkins reported a normal TcTU mean±SD of 1.73±0.85% and a normal TcTU range of 0.5 to 4% in 1971. 3 Another study found a normal TcTU range of 0.4 to 2.5%. 6 A normal TcTU mean of 0.9% and a normal TcTU range of 0.1 to 6.3% were reported in another study. 1 On the other hand, one study found that the normal TcTU range was 0.4 to 1.7%. 7 The fact that ^99m^ Tc pertechnetate has a wide physiological distribution in the body, including gastric mucosal uptake and excretion, salivary gland uptake and excretion, kidney uptake and excretion, and blood pool stagnation, could explain the low normal TcTU. As a result, a small amount of ^99m^ Tc pertechnetate is left to be taken up by the thyroid gland.

TcTU overlap between normal and thyrotoxic patient groups is regarded as a drawback of ^99m^ Tc pertechnetate in thyroid uptake evaluation. Because the normal range of TcTU is generally low and narrow, this disadvantage is exaggerated. 8 9 In agreement with this, our data revealed that 31% of our thyrotoxic patients had normal TcTU values (overlapped with normal range). This is in line with previous research, which found that 8 of 26 (31%) thyrotoxic patients had normal TcTU values. 4 In another study, 24 of 201 Graves' disease patients (12%) had normal TcTU values. 6 TcTU values in 57 Graves' disease patients were also found to range from 0.97 to 40.1%. 10 However, Graves'/toxic nodular goiter was well visualized even with normal TcTU values, whereas thyroid gland was nonvisualized or poorly visualized in subacute thyroiditis, regardless of how low the TcTU value was.

Several factors might influence the thyroidal trapping of ^99m^ Tc pertechnetate, which could explain why some hyperthyroid patients have normal TcTU values. A high load of a stable iodine competes with ^99m^ Tc trapping in the thyroid gland and ^99m^ Tc reabsorption in the kidneys, resulting in less ^99m^ Tc trapping in the thyroid gland and more ^99m^ Tc excretion in the kidneys. This is seen in patients who have had an iodinated contrast enhanced computed tomography scan recently. Because there is continuous ^99m^ Tc exchange between the thyroid gland and the plasma, large extracellular volume, such as in edematous patients or anasarca, results in less ^99m^ Tc trapping in the thyroid. Renal failure may affect ^99m^ TcTU because renal ^99m^ Tc clearance accounts for 30% of normal ^99m^ Tc clearance from the human body, leaving more ^99m^ Tc available for thyroid trapping. Because ^99m^ Tc is partially bound to albumin, the amount of unbound free ^99m^ Tc available for thyroid trapping may have an indirect effect on thyroid uptake. As a result, thyroid ^99m^ Tc trapping may be affected by serum albumin levels. Antithyroid medications may prevent ^99m^ Tc from being trapped in the thyroid in the same way that they prevent iodine from being trapped. 11

Our research demonstrates the utility of ^99m^ Tc pertechnetate in thyrotoxic patients, who will benefit from the use of ^99m^ Tc pertechnetate as a tracer in TcTU and scans as previously mentioned. 1 This method has been found to be useful in our department because ^131^ I is readily available for imaging rather than ^123^ I, which is expensive and has a short half-life. We were able to get rid of ^131^ I from our patients using this method. At the same time, we were able to diagnose most of our patients accurately by combining qualitative and quantitative image analyses to overcome the limitations of TcTU's narrow range, which can result in falsely normal TcTU values that overlap with the normal TcTU range. We believe that in most developing countries where ^123^ I is not readily available, this approach will be beneficial.

Because of the high radiation burden on the patient's thyroid gland, ^131^ I abandonment is clinically significant for our patients. For the same administered activity, the dose to the thyroid from ^131^ I is approximately 100 times greater than that from ^123^ I (∼ 1 rad/μCi [10 mGy/0.037 MBq] versus 1 rad/100 μCi [10 mGy/3.7 MBq]). The absorbed thyroid dose from ^99m^ Tc pertechnetate is approximately 1 rad/5,000 μCi (10 mGy/185 MBq). 12 The average dose given to the thyroid gland is 5 mCi (185 MBq) ^99m^ Tc, which results in 1 rad of radiation absorption. When compared with the radiation absorbed from radioiodine, this is a negligible dose.

## Conclusion

Our local normal TcTU mean and range were found to be similar to those previously published. In our laboratory, ^99m^ Tc pertechnetate was used to functionally evaluate thyrotoxic patients in North Jordan as an alternative to radioiodine. The TcTU test was found to be useful in the evaluation of thyrotoxic patients in our retrospective study. In approximately 70% of cases, the test results were accurate. The diagnosis was almost reached in the remaining 30% of patients by combining quantitative and qualitative image features. Because ^131^ I is readily available in our country and ^123^ I is not, we were able to eliminate ^131^ I from our daily practice, resulting in a significant reduction in radiation burden in our patients.

## References

[1] Macauley M Shawgi M Ali T Assessment of normal reference values for thyroid uptake of technetium-99m pertechnetate in a single centre UK population Nucl Med Commun 2018 39 09 834 838 29877994 10.1097/MNM.0000000000000876

[2] Al-Muqbel K M Tashtoush R M Patterns of thyroid radioiodine uptake: Jordanian experience J Nucl Med Technol 2010 38 01 32 36 20159926 10.2967/jnmt.109.069146

[3] Atkins H L Technetium-99m pertechnetate uptake and scanning in the evaluation of thyroid function Semin Nucl Med 1971 1 03 345 355 4329842 10.1016/s0001-2998(71)80007-7

[4] Sucupira M S Camargo E E Nickoloff E L Alderson P O Wagner H N Jr The role of 99mTc pertechnetate uptake in the evaluation of thyroid function Int J Nucl Med Biol 1983 10 01 29 33 6305864 10.1016/0047-0740(83)90030-x

[5] Uchida T Suzuki R Kasai T Cutoff value of thyroid uptake of (99m)Tc-pertechnetate to discriminate between Graves' disease and painless thyroiditis: a single center retrospective study Endocr J 2016 63 02 143 149 26581846 10.1507/endocrj.EJ15-0441

[6] Ikekubo K Hino M Ito H Thyrotoxic Graves' disease with normal thyroidal technetium-99m pertechnetate uptake Ann Nucl Med 1990 4 02 43 48 2171608 10.1007/BF03164594

[7] Ramos C D Zantut Wittmann D E Etchebehere E C Tambascia M A Silva C A Camargo E E Thyroid uptake and scintigraphy using 99mTc pertechnetate: standardization in normal individuals Sao Paulo Med J 2002 120 02 45 48 11994772 10.1590/S1516-31802002000200004 PMC11146234

[8] Maisey M N Natarajan T K Hurley P J Wagner H N Jr Validation of a rapid computerized method of measuring 99mTc pertechnetate uptake for routine assessment of thyroid structure and function J Clin Endocrinol Metab 1973 36 02 317 322 4566719 10.1210/jcem-36-2-317

[9] Schneider P B Simple, rapid thyroid function testing with 99mTc-pertechnetate thyroid uptake ratio and neck/thigh ratio AJR Am J Roentgenol 1979 132 02 249 253 105593 10.2214/ajr.132.2.249

[10] Kidokoro-Kunii Y Emoto N Cho K Oikawa S Analysis of the factors associated with Tc-99m pertechnetate uptake in thyrotoxicosis and graves' disease J Nippon Med Sch 2006 73 01 10 17 16538017 10.1272/jnms.73.10

[11] Hays M T Berman M Pertechnetate distribution in man after intravenous infusion: a compartmental model J Nucl Med 1977 18 09 898 904 893788

[12] Mettler F A Jr. Guiberteau M J Essentials of Nuclear Medicine Imaging 6th edition. Saunders Philadelphia, PA 2012

